# Prognostic analysis of inflammatory response-related genes and biomarkers in patients with urothelial carcinoma of ureter

**DOI:** 10.3389/fgene.2023.1139412

**Published:** 2023-03-02

**Authors:** Huaian Chen, Shuo Liu, Xiujun Li, Zhe Wang, Chao Zhang

**Affiliations:** First Affiliated Hospital of Hebei North University, Zhangjiakou, Hebei, China

**Keywords:** patients with urothelial carcinoma of ureter, inflammatory reaction, related genes, prognostic analysis, image fusion

## Abstract

Ureteral urothelial carcinoma is a common urinary system tumor, accounting for 40% to 60% of all ureteral diseases. This study attempted to analyze the prognosis of patients with urothelial carcinoma, judging ureteral urothelial carcinoma by genes and biomarkers of inflammatory response. In this paper, co-expression network analysis and gene-based image fusion evaluation methods were proposed to obtain the prognosis results of patients with ureteral urothelial carcinoma. The experimental results showed that the levels of PLR and NLR increased, and the levels of HGB and HCT decreased; high PLR and high NLR levels, low HGB and low HCT levels were all risk factors affecting bladder urothelial carcinoma, and their ratios (OR) were 1.023, 1.611, 0.961, 0.859, 1.015, 1.072, 0.979, and 0.951, respectively. However, high PLR and high NLR levels were independent risk factors for bladder urothelial carcinoma, and their OR values were 1.497 and 1.071, respectively. Through biomarker diagnosis, the area under the curve, sensitivity, specificity and Youden index of hsa-mir-17, hsa-mir-93, hsa-mir-429 and hsa-mir-20a all exceeded 0.9, indicating that this is a potential diagnostic indicators. All in all, during the treatment of ureteral cancer, in order to reduce tumor recurrence, systemic therapy should be combined with ureteral cancer. In addition, this study also analyzed the prognosis of chemotherapy patients, and the results showed that immunotherapy may increase the risk of tumor cell reperfusion during chemotherapy.

## 1 Introduction

Epithelial carcinoma of the urinary tract is a cancer that occurs in some epithelium covering the urinary tract. If all covered epithelial tissues are exposed to urine for a long time, it is very likely to be affected by carcinogenic factors in urine to form cancer. Although urothelial carcinoma and cystourethropathy have the same cell origin, the two have completely different biological manifestations and prognosis. Upper tract urothelial carcinoma is a relatively rare heterogeneous cancer, but the incidence of upper tract urothelial carcinoma in China has increased rapidly in recent years, and the number of related reports has gradually increased. In the nearly 30 years from 1973 to 2005, the overall incidence of upper urinary tract urothelial carcinoma in China has increased steadily, which is mainly related to the increase of ureteral carcinoma. Ureteral carcinoma used to be one of the rarest urological neoplasms, accounting for only about one percent of all urothelial malignancies. In recent years, due to the improvement of detection methods and the growth of people’s lives, the incidence rate is also increasing. The prognosis of ureteral cancer is poor, and the five-year survival rate is about 41%–67%. This is mainly related to the thinner wall of the ureter, the richer lymphatic drainage near it, the easy penetration of cancer cells through the muscle layer, and the invasion and movement that can be caused. Although there are various treatment methods for urothelial carcinoma of the ureter, in general, the 10-year survival rate of most patients after diagnosis is still very low, and only about 20%–30% of patients can survive for more than 10 years. Most of these patients have a poor prognosis, and their 5-year survival rate is only 15%. Among tumor-associated inflammatory response pathway genes, about 50% of the data were concentrated in patients with urothelial carcinoma. Therefore, studying and analyzing the expression of inflammation-related genes associated with ureteral carcinoma is of great significance for determining the prognosis of patients. This article analyzes the prognosis of patients with urothelial carcinoma of the ureter through inflammatory response genes and biomarkers in order to make certain contributions to the treatment of urothelial carcinoma of the ureter.

Based on the existing research results, various scholars have carried out relevant research on Urothelial Carcinoma of Ureter: Ureteral fibroepithelial polyp is a rare benign lesion, and it is a good diagnostic index. Bansal Devanshu reported a case of severe hematuria in a 56-year-old woman. After a preliminary examination, she was diagnosed with lower ureteral carcinoma and underwent surgery (Bansal et al., 2022). Small cell carcinoma is a common type of neuroendocrine cancer, mostly found in the lung, accounting for about 20%–30% of all lung cancers. A few are outside the lung, accounting for about 0.1%–0.4% of all small cell carcinomas. Extrapulmonary small cell carcinoma may appear in multiple organs of the body, while small cell carcinoma of the urinary tract is not uncommon in clinical practice. However, sporadic case reports have also been reported in recent years, and primary small cell carcinoma of the ureter is even rarer. Yang et al. (2019) studied the cases of patients with primary ureteral small cell carcinoma after kidney transplantation, and then the other side of the ureter and bladder with primary urothelial carcinoma. The purpose of Alessandro et al. (2020) is to explore the clinical diagnosis and prognostic value of upper tract urothelial carcinoma. A total of 6,619 cases were identified in the surveillance, epidemiology and final result prevalence database, including 3,719 cases of renal pelvis involvement and 2,971 cases of ureteral involvement. Alessandro et al. (2020) evaluated predictors of surgical technique. However, these scholars lacked certain technical demonstrations on the exploration of urothelial ureteral carcinoma. After research, it was found that genes related to inflammatory response have certain research value on the prognosis of urothelial ureteral carcinoma. For this, relevant literature on inflammatory response was consulted.

Some scholars also have some research on inflammatory response: Mori et al. (2021) explored the prognostic role of preoperative systemic immune inflammatory index in patients with upper tract urothelial carcinoma undergoing radical nephroureterectomy. Itami et al. (2019) evaluated the clinical relevance of a comprehensive preoperative assessment of inflammatory, nutritional, and muscle markers in patients with upper tract urothelial carcinoma undergoing therapeutic nephroureterectomy. Chen et al. (2021). evaluated the significance of indicators related to inflammation in predicting the prognosis of ureteral carcinoma. However, these scholars did not analyze the prognosis of patients with Urothelial Carcinoma of Ureter based on inflammatory response-related genes and biomarkers, but only discussed it from a superficial level.

In this paper, the following conclusions are drawn by analyzing the prognosis of patients with Urothelial Carcinoma of Ureter: Ureteral cancer should be combined with systemic therapy to reduce its recurrence rate. On this basis, it is necessary to conduct an in-depth discussion on its related genes and its influence on the recurrence rate and prognosis of ureteral tumors.

The innovations of this paper are as follows: 1) To explore the detection methods of ureteral cancer, study the gene co-expression network analysis and propose an image fusion evaluation method; 2) Based on this, the simulation analysis of the prognosis experiment of patients with urothelial carcinoma of the ureter was carried out.

## 2 Prognostic analysis method of inflammatory response-related genes in patients with urothelial carcinoma of ureter

### 2.1 Detection and prognostic analysis of ureteral carcinoma

**FIGURE 1:**
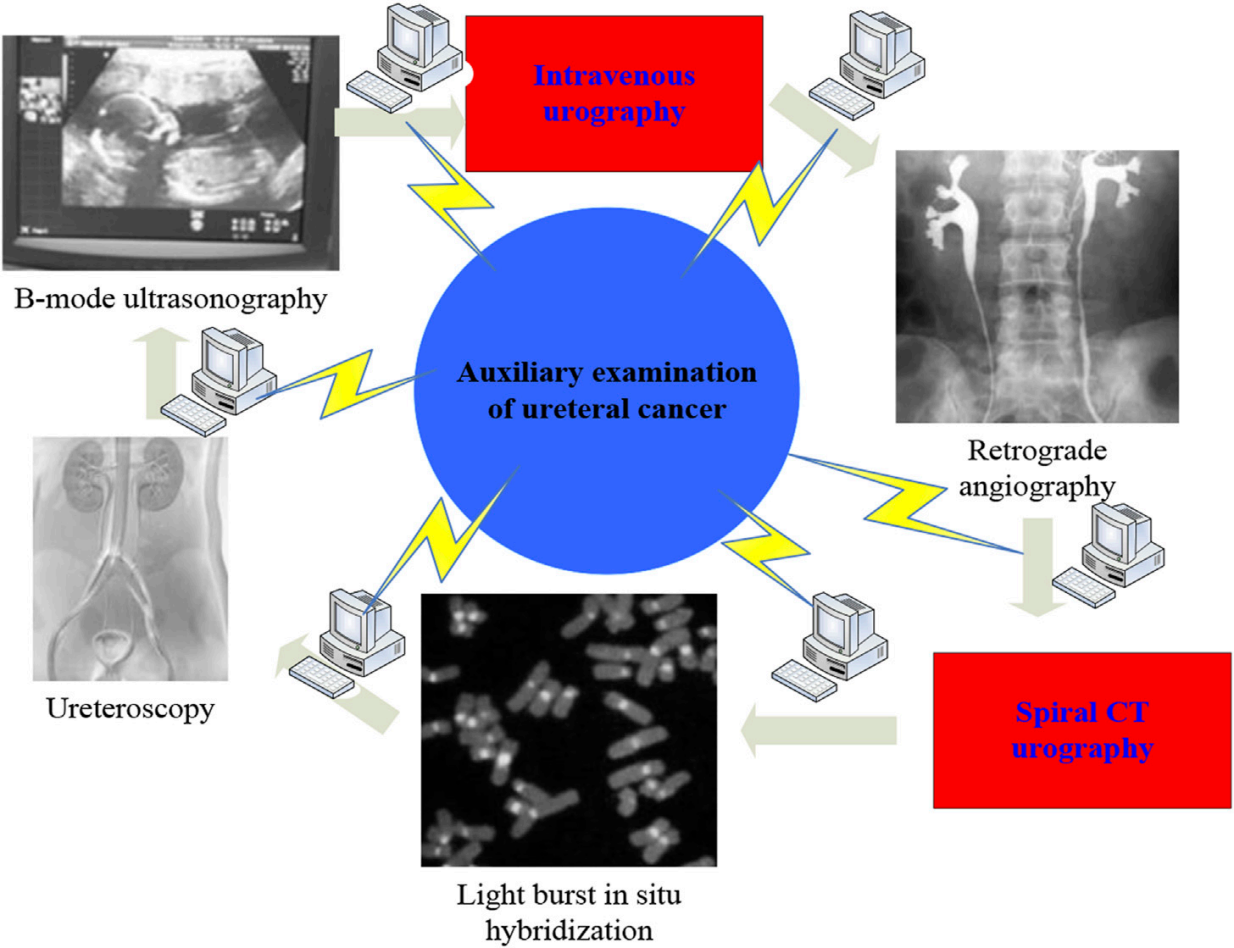
Auxiliary examination for ureteral cancer.

The preoperative diagnosis of ureteral carcinoma mainly relies on imaging, endoscopy and histopathological examination. At present, there are mainly B-ultrasound, intravenous urography (IVU), retrograde radiography, spiral CT urography (CTU), fluorescence *in situ* hybridization (FISH), ureteroscopy, etc. (Mounsif et al., 2019; Petros et al., 2021). Ureteral tumor refers to the tumor occurring in the ureter, which can be divided into benign tumor and malignant tumor according to its nature. Figure 1 shows the auxiliary examination for ureteral cancer. In the past, the auxiliary examination of ureteral cancer mostly relied on IVU and retrograde angiography, but due to the development of imaging and imaging techniques, such as CTU, urologists can more directly observe ureteral tumors (Wang et al., 2018; Takashi et al., 2020). CTU has advantages that other auxiliary examinations do not have: 1) In coronal and sagittal three-dimensional reconstruction of the urinary tract, it can comprehensively and intuitively display the entire tortuous course of the upper urinary tract, greatly increasing the diagnostic accuracy of renal and urothelial malignant tumors. 2) Compared with IVU, CTU has a diameter of less than 15 mm and a mass that is covered by the superposition of gas. Therefore, the diagnostic sensitivity and accuracy of CTU for ureteral transitional cell carcinoma can be as high as 89%–100%. 3) CTU can not only show tumors growing on the surface of the ureter, but also infiltrating and growing tumors, and even lead to stenosis and atresia of the ureteral lumen, which is not available in B-ultrasound and IVU (Wang et al., 2019; Ren et al., 2021). 4) CTU can display fat tissue around the renal pelvis and ureter, enlarged lymph nodes, distant metastasis, etc., and can make a correct diagnosis of clinical staging, laying the foundation for preoperative evaluation. 5) CTU is divided into arterial phase, venous phase, and excretion phase. Compared with other auxiliary examinations, more imaging data can be obtained, which is helpful to improve the accuracy of clinical diagnosis. Spiral CT urography is a continuous volume scan performed during the peak period of contrast agent excretion by injecting contrast agent. The obtained image is processed by computer to obtain a three-dimensional representation of the urinary tract. CTU has become an important means of diagnosing ureteral tumors.

Changes in lifestyle such as smoking and drinking are the main factors affecting the prognosis of patients with ureteral cancer (Chang et al., 2021; Zhou et al., 2022). The survival analysis of patients with ureteral cancer found that the prognosis of smokers was worse than that of women (Sebastiano et al., 2019; Teruo et al., 2020). The study also found that there were certain differences in lifestyle changes of patients with ureteral cancer at different stages. In conclusion, we can see from the influence of the above factors that we should pay attention to the prognostic evaluation of ureteral cancer in clinical practice.

Because the age of the incidence of ureteral cancer is younger in clinical practice, and the location of the incidence is mostly between the bladder and the kidney, early diagnosis and treatment effect are very important (Nir et al., 2020). Clinically, it is necessary to comprehensively analyze and evaluate the tumor stage and pathological classification, the relationship between stage and clinical prognosis, and the prognosis between different stages, so as to obtain the best treatment plan suitable for the patient’s condition. In addition, for the elderly patients with ureteral cancer or those with family history and early onset of ureteral cancer, surgery or chemotherapy should be carried out as far as possible to avoid death due to deterioration of the condition. Since the incidence rate of ureteral cancer ranks first among the malignant tumors of the urinary system, it is very important to choose the treatment strategy for this disease.

### 2.2 Gene co-expression network analysis

Since the data of the network module is easily disturbed by outlier data, it is important to ensure the reliability of the results of the subsequent gene co-expression system network analysis. First of all, the information must be preprocessed to control the quality of the data, so all outlier sample information must be cleared before the establishment of the gene co-expression system. The inter-array correlation (IAC) coefficient plot can be used to assess the spread of chip data, the sample clustering dendrogram, the IAC distribution line graph, and the average IAC scatter point curve to evaluate the degree of data outlier can also be used, and the data that is obviously scattered should be deleted.

WGCNA (Weighted Gene Co-Expression Network Analysis) is an Internet technical analysis package for weighted genome co-expression, which can be used to discover template resources and module hub genomes in chip resources. In this package, concepts such as module, eigenvector gene, module identity, gene significance, module significance, hub gene, etc. are defined. The template refers to a group of genomes with similar expression profiles, which can indicate that their genomes are highly related in various functions. The characteristic vector genome refers to the first principal component genome that can represent the expression levels of all genes in a module. The template identity refers to the similarity between the expression profile of each gene and a characteristic vector gene expression profile. The significance of the genome refers to the relative value of the *p*-value of the significance test of the differential expression of the genome between a genome and different groups of clinicopathological characteristics, which is used to reflect the degree of correlation between the genome and external signals. The significance of the template refers to the average of the gene significance of each gene group involved in the module, which can indicate the high correlation between the module and some clinical application signals. The central genome refers to a group of key genes with the largest connection in a certain model, and it can also be called the most important gene group in a certain model. It determines the important characteristics of the template to a certain extent, and the hub genome in establishing and describing the network model is usually more biologically meaningful.

### 2.3 Image fusion evaluation

Medical imaging, also known as image acquisition, image conversion, image visualization, etc., refers to the use of image scanners to obtain image data non-invasively, and to provide information such as contrast, brightness, and details of images based on image technology. Current medical imaging acquisition modes include: ultrasound, computed tomography (CT), magnetic resonance imaging (MRI), positron emission computed tomography (PET), single photon emission computed tomography (SPECT), etc.

CT images use attenuation coefficients to measure tissue density, which is an important anatomical data. CT images show that the bone image is clear, so the bones and contrast-enhanced blood vessels can be clearly distinguished. However, its performance on soft tissue lesions is limited due to the high cost of high-density *in vivo* detection, which may adversely affect its molecular imaging. MRI is the rotational imbalance produced by the hydrogen protons in the human body in a strong magnetic field. This technology can accurately display the blood perfusion of different tissues, and can clearly reflect the imaging features of soft tissues. However, its gray level distribution is still non-uniform. At the same time, due to the different types of scanning equipment in different regions, there are certain differences in their intensity values. Both PET and SPECT are imaging techniques for visualizing radioactive material. PET imaging can be performed over a dynamic range, for example, in temporal sequences of two-dimensional images of active regions of the brain. SPECT uses a narrow-span collimator, and its imaging has high spatial resolution, but it lacks in sensitivity compared with PET.

**FIGURE 2:**
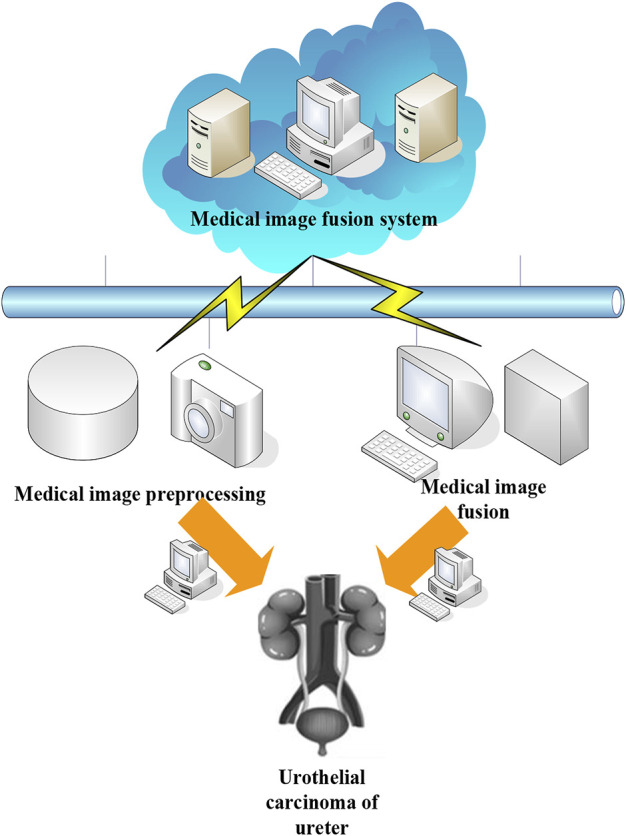
Application of medical image fusion in Urothelial Carcinoma of Ureter.

In order to further treat patients with urothelial carcinoma of the ureter, it is necessary to analyze the images of the patient’s site. The subjective evaluation refers to the analysis of the advantages and disadvantages of the fusion algorithm, such as image distortion, internal details, etc., using the researcher’s reasoning. In terms of image fusion, the subjective evaluation method is simple and reliable. However, due to the influence of the surrounding environment, there are great differences in people’s mental state and vision level, so the evaluation accuracy of the image is not high. For this reason, researchers judge the quality of image fusion on the basis of comprehensive evaluation and objective evaluation. Figure 2 shows the application of medical image fusion in Urothelial Carcinoma of Ureter.

At present, in order to test the clarity and validity of images output by urothelial carcinoma of the ureter, scholars have proposed many indicators of image parameters. For the above evaluation indicators, some representative evaluation indicators can be selected. The public variables included in the following formulas are as follows: $O_{G}$ is the fused image, and $O_{T}$ is the reference image, and Z and M are the width and height of the image respectively.

#### 2.3.1 Entropy (EN)

As EN increases, the information in the fused image also increases. EN can be represented by the following formula.

$$EN=-\overset{A-1}{\underset{o=0}{\sum }}q\left(o\right){\log\;}_{2}q\left(o\right)$$ (1)

where o is the gray level and $q\left(o\right)$ is the probability associated with o.

#### 2.3.2 Gradient index (W)

The output range of W is between [0,1]. When the output is close to 1, clear image boundary information can be obtained. W can be represented by the following formula.

$$W=\frac{\overset{Z}{\underset{o=1}{\sum }}\overset{M}{\underset{k=1}{\sum }}\left(W_{O_{1}O_{G}}\left(o,k\right)E_{O_{1}}\left(o,k\right)+W_{O_{2}O_{G}}\left(o,k\right)E_{O_{2}}\left(o,k\right)\right)}{\overset{Z}{\underset{o=1}{\sum }}\overset{M}{\underset{k=1}{\sum }}\left(E_{O_{1}}\left(o,k\right)+E_{O_{2}}\left(o,k\right)\right)}$$ (2)

where E is a sliding window with a fixed size.

#### 2.3.3 Average gradient (AVG)

The larger the AVG value, the better the quality of the fused image.

$$AVG=\frac{1}{ZM}\overset{Z}{\underset{o=1}{\sum }}\overset{M}{\underset{k=1}{\sum }}\sqrt{\frac{\nabla O_{G_{c}}^{2}\left(o,k\right)+\nabla O_{G_{u}}^{2}\left(o,k\right)}{2}}$$ (3)

where $\nabla O_{G_{c}}^{2}\left(o,k\right)$ is the first-order difference of $O_{G}$ in the c direction, and $\nabla O_{G_{u}}^{2}$ is the first-order difference of $O_{G}$ in the u direction.

#### 2.3.4 Mutual information (MI)

MI refers to the further extraction of information such as image details and textures based on the intensity similarity between the $O_{T}$ image and the $O_{G}$ image. MI can be represented by the following formula.

$$MI=\overset{Z}{\underset{o=1}{\sum }}\overset{M}{\underset{k=1}{\sum }}j_{O_{T}O_{G}}\left(o,k\right)\times {\log\;}_{2}\left(\frac{j_{O_{T}O_{G}}\left(o,k\right)}{j_{O_{T}}\left(o,k\right)j_{O_{G}}\left(o,k\right)}\right)$$ (4)

where $j_{O_{T}O_{G}}$ is the joint gray level histogram of $O_{T}$ and $O_{G}$ .

#### 2.3.5 Standard deviation (SD)

The value of SD is proportional to the contrast of the fused image. SD can be represented by the following formula.

$$SD=\sqrt{\frac{1}{ZM}\overset{Z}{\underset{o=1}{\sum }}\overset{M}{\underset{k=1}{\sum }}{\left(O_{G}\left(o,k\right)-i_{O_{G}}\right)}^{2}}$$ (5)

where, i is the average value of the image.

#### 2.3.6 Spatial frequency (SF)

The SF value is proportional to the overall clarity of the fused image, and is calculated from the row frequency and column frequency. SF can be represented by the following formulas.

$$SF=\sqrt{{RF}^{2}+{CF}^{2}}$$ (6)

$$RF=\sqrt{\frac{1}{ZM}\overset{Z}{\underset{o=1}{\sum }}\overset{M}{\underset{k=1}{\sum }}{\left(O_{G}\left(o,k\right)-O_{G}\left(o,k-1\right)\right)}^{2}}$$ (7)

$$CF=\sqrt{\frac{1}{ZM}\overset{Z}{\underset{o=1}{\sum }}\overset{M}{\underset{k=1}{\sum }}{\left(O_{G}\left(o,k\right)-O_{G}\left(o-1,k\right)\right)}^{2}}$$ (8)

Therefore, the above indicators can be used to analyze the effect of Urothelial Carcinoma of Ureter images very well.

## 3 Prognosis results of patients with urothelial carcinoma of ureter

### 3.1 Experimental materials and methods

This article collected a total of 200 patients with Urothelial Carcinoma of Ureter who were treated in the Department of Urology of Hospital, First Affiliated Hospital of Hebei North University from June 2018 to December 2021 and were hospitalized for surgical treatment and had clear pathological diagnosis after surgery. Among them, 120 were male, accounting for 60% of the total number of cases, and 80 were female patients, accounting for 40% of the total number of cases. The age of onset of patients included in the case data was 38–87 years old, 48 patients (24%) were 50–59 years old, 65 patients (32.5%) were 60–69 years old, and 87 patients (43.5%) were over 70 years old. The results showed that upper urothelial carcinoma increased with age, as shown in Table 1.

**TABLE 1:** Experimental object data.

Gender	Number of people	Proportion
Male	120	60%
Female	80	40%
Age	Number of people	Proportion
50–59 years old	48	24%
60–69 years old	65	32.5%
Over 70	87	43.5%

This paper studied the changes of platelet-lymphoid ratio (PLR), neutrophil-lymphocyte ratio (NLR), hemoglobin (HGB), and hematocrit (HCT) in patients with bladder urothelial carcinoma before operation, so as to study the clinical significance of relevant indicators. Their impact on bladder urothelial carcinoma, its direct relationship between clinicopathological grade and stage, and its clinical utility were analyzed.

Statistical analysis tools: All statistical analysis uses SPSS19.0 statistical analysis software package. *p* < 0.05 means significant difference, and the difference is statistically significant.

### 3.2 Inflammatory indicators and risk factors associated with urothelial carcinoma of ureter

**FIGURE 3:**
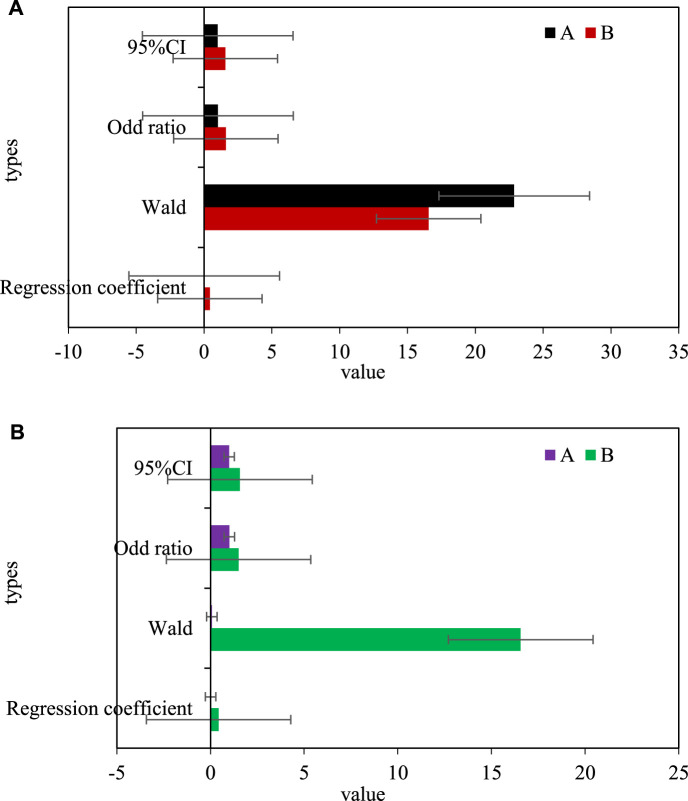
**(A)** Univariate logistic risk factor analysis of inflammatory markers and ureteral urothelial carcinoma. **(B)** Multivariate logistic risk factor analysis of inflammatory indicators and ureteral urothelial carcinoma.

Using different dependent variables of serum PLR (A) and NLR (B) levels, the incidence rate of bladder urothelial carcinoma was used as a dependent variable, and the univariate Logistic risk factor study was first used. The results showed that: Increased levels of PLR and NLR are the main risk factors for bladder urothelial carcinoma, with odd ratio (OR) values of 1.023 and 1.611, and 95% confidence intervals (CI) of 1.009 and 1.569, respectively. Then, based on the conclusion of the single-factor Logistic risk factor study, the indicators with statistical value (PLR, NLR) in the single-factor analysis were introduced into the multi-factor Logistic risk factor study, and the risk factors of bladder urothelial carcinoma with independent value was screened out. The results showed that high NLR content was an independent risk factor for bladder urothelial carcinoma, with an OR value of 1.497 and a 95% CI of 1.573. As shown in Figure 3, Figure 3A is the univariate Logistic risk factor analysis of inflammatory indicators and Urothelial Carcinoma of Ureter, and Figure 3B is the multivariate Logistic risk factor analysis of inflammatory indicators and Urothelial Carcinoma of Ureter.

### 3.3 Hemoglobin index and related risk factors of urothelial carcinoma of ureter

**FIGURE 4:**
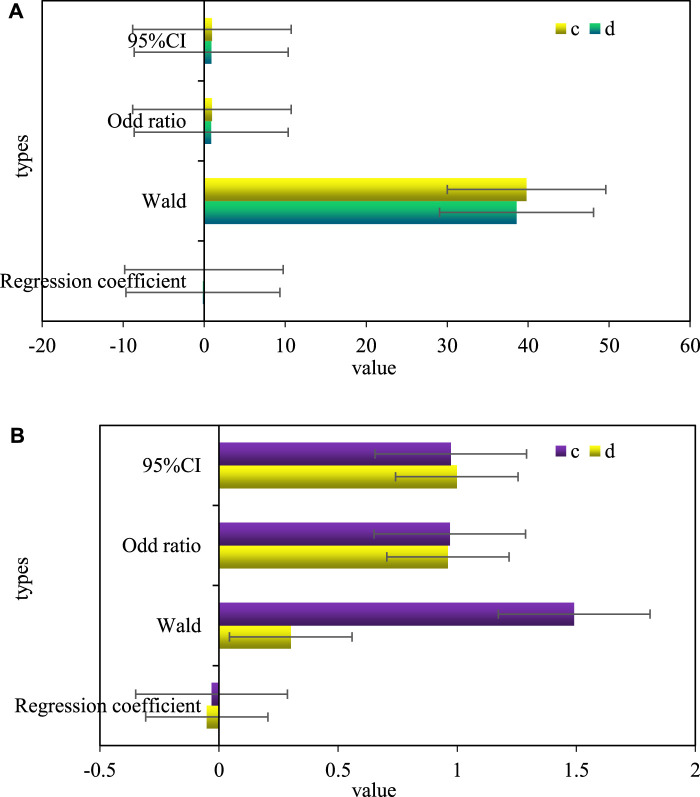
**(A)** Univariate logistic risk factor analysis of hemoglobin index and ureteral urothelial cancer. **(B)** Multivariate logistic risk factor analysis of hemoglobin index and ureteral urothelial carcinoma.

The different dependent variables of HGB (c) and HCT (d) levels in serum and the incidence rate of Urothelial Carcinoma of Ureter were used as dependent variables, and single-factor Logistic risk factors were used for research. The results showed that the decline of HGB and HCT levels were the main risk factors affecting the carcinogenesis of ureteral urothelium, the OR values were 0.961 and 0.859, and the 95% CI were 0.961 and 0.873. Based on the conclusions of the single-factor Logistic risk factor study, the factors with statistical value (HGB, HCT) in the single-factor analysis were introduced into the multi-factor Logistic risk factor study, and the risk factors with independent value that caused ureteral urothelial canceration was screened out. As shown in Figure 4, Figure 4A is a univariate Logistic risk factor analysis of hemoglobin index and Urothelial Carcinoma of Ureter, and Figure 4B is a multivariate Logistic risk factor analysis of hemoglobin index and Urothelial Carcinoma of Ureter.

### 3.4 Risk factors associated with inflammatory indicators and pathological grading of urothelial carcinoma of ureter

**FIGURE 5:**
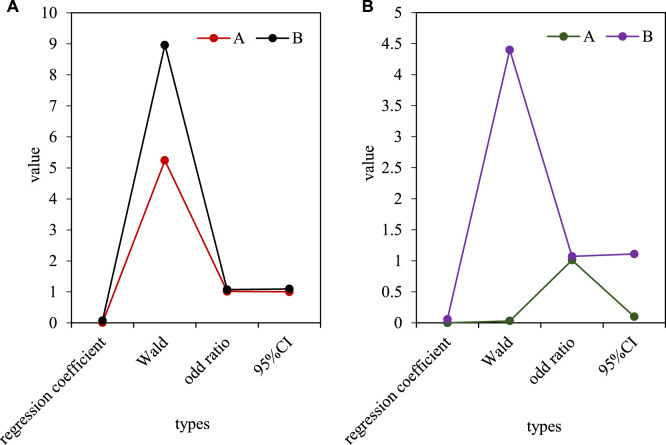
**(A)** Univariate logistic risk factor analysis of inflammatory indicators and pathological grades in ureteral urothelial carcinoma. **(B)** Multivariate logistic risk factor analysis of inflammatory index and pathological grade in ureteral urothelial carcinoma.

Taking the difference in serum PLR (A) and NLR (B) levels as the dependent variable, and the pathological classification of Urothelial Carcinoma of Ureter as the dependent variable, this paper first used a single-factor Logistic risk factor study. The results showed that high PLR and high NLR content were the main risk factors leading to the pathological classification of Urothelial Carcinoma of Ureter, with OR values of 1.015 and 1.072, and 95% CI of 1.003 and 1.096, respectively. Based on the results of single factor Logistic risk factor analysis, the indexes (PLR, NLR) with statistical significance in single factor analysis were included in the multivariate Logistic risk factor analysis. Risk factors with independent meanings that were closely related to the classification of ureteral and urothelial carcinoma lesions were selected. The results showed that high NLR level was an independent risk factor affecting the risk of pathological classification of bladder urothelial carcinoma, with an OR value of 1.071 and a 95% CI of 1.111. As shown in Figure 5, Figure 5A is a single factor Logistic risk factor analysis of the inflammation index and the pathological grade of Urothelial Carcinoma of Ureter, and Figure 5B shows the multifactor Logistic risk factor analysis of inflammation index and pathological grade of Urothelial Carcinoma of Ureter.

### 3.5 Risk factors related to hemoglobin index and pathological grade of urothelial carcinoma of ureter

**FIGURE 6:**
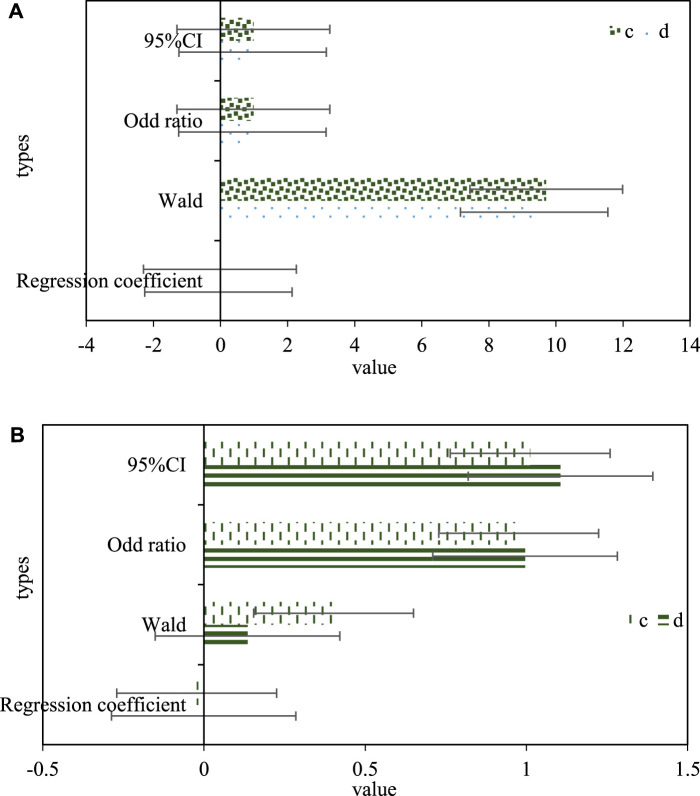
**(A)** Univariate logistic risk factor analysis of hemoglobin index and pathological classification for ureteral urothelial carcinoma. **(B)** Multivariate logistic risk factor analysis method for hemoglobin index and pathological type of ureteral urothelial carcinoma.

Serum low HGB (c) and low HCT (d) levels were designed as independent variables, and the pathological grade of Urothelial Carcinoma of Ureter was designed as dependent variables. First, through the single factor Logistic risk factor analysis, the results showed that: Low HGB and low HCT levels were risk factors affecting the pathological grade of Urothelial Carcinoma of Ureter, with OR values of 0.979 and 0.951, and 95%CI of 0.983 and 0.956, respectively. Based on the research results of the single factor Logistic risk factor analysis method, the factors (HGB, HCT) with statistical significance in the single factor analysis were introduced into the multifactor Logistic risk factor analysis method. Thus, risk factors with independent meanings that are closely related to the classification of ureteral and urothelial carcinoma lesions were screened out. The results showed that there was no single risk factor in HGB and HCT that reflected the risk of pathological classification of urothelial carcinoma of the ureter. As shown in Figure 6, Figure 6A is a univariate Logistic risk factor analysis of hemoglobin index and pathological classification of Urothelial Carcinoma of Ureter. Figure 6B shows the multifactor Logistic risk factor analysis method of hemoglobin index and pathological classification of urothelial carcinoma of the ureter.

### 3.6 Evaluation of the diagnostic effect of biomarkers

**FIGURE 7:**
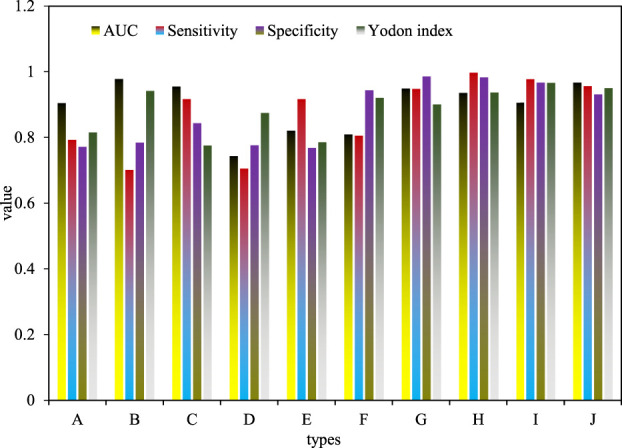
Results of ROC curve analysis of expressed genes.

A better marker for tumor detection, the closer the area under the receiver operating characteristic curve (ROC), sensitivity, specificity, and Youden index are to one, the stronger it is. It is generally considered that the detection ability of the test markers with the area under the ROC curve, sensitivity, specificity and Youden index all exceeding 0.9 is strong, and it is suitable for clinical diagnosis. In this paper, ROC curve research was carried out on ten newly screened expressed genes, and the capacity under the curve (AUC), sensitivity, specificity and Youden index were calculated, and the results are shown in Figure 7. At the same time, taking into account the above technical indicators, it was found that the area under the ROC curve, sensitivity, specificity and Youden index are higher than 0.7, suggesting that it may be of general diagnostic ability as a biomarker for urothelial carcinoma of the ureter, and has certain reference value. The range, sensitivity, specificity and Youden index of Hsa-mIr-17 (G), hsa-mir-93 (H), hsa-mir-429 (I), hsa-mir-20a (J) curves are all higher than 0.9, indicating that its detection function is very good, and it has become a potential detection marker for bladder urothelial carcinoma.

## 4 Conclusion

Urothelial Carcinoma of Ureter, as the most common malignant tumor in the urinary system, has attracted more and more attention. Its occurrence and development is a complex pathological change process, which is affected by many factors. The molecular mechanism and specific therapeutic targets related to its occurrence and development have always been the hotspots in the field of medical research. With the development of molecular ecology of cancer genes, a series of studies on large-scale proteomics and genomics have shown that the malignant development, invasion, transformation and sociobiological processes of bladder cancer are closely related to the different expressions of mRNA and miRNA. The theoretical research results played a very critical role in finding biomarkers of bladder urothelial carcinoma and important gene molecular targets that directly affect the pathological process of bladder urothelial carcinoma. During the treatment of ureteral cancer, it is necessary to consider the use of targeted and immunotherapy in combination with systemic therapy to reduce cancer recurrence and also need to take into account the prognosis of chemotherapy patients. It showed that the use of immunotherapy during chemotherapy can increase the risk of recurrent infusion of tumor cells when chemotherapy drugs are infused. This paper also found that inflammation-related genes have higher expression levels in patients with RCC, indicating that inflammatory response-related pathways may be an important factor in the development of RCC. In the future, this paper needs to further study the genes involved in the inflammatory response pathway and the significance of predicting the postoperative recurrence rate and prognosis of ureteral cancer. However, due to the limitations of time and technology, this paper has not carried out a detailed study of the problems encountered in the study of ureterourethral epithelial carcinoma, which will be further discussed in the future.

## Data Availability

The original contributions presented in the study are included in the article/supplementary material, further inquiries can be directed to the corresponding author.

## References

[B1] Alessandro V. Antonelli A. Martini A. Falagario U. Carrieri G. Grob M. B. (2020). Ureteral location is associated with survival outcomes in upper tract urothelial carcinoma: A population‐based analysis. Int. J. Urology 2711, 966–972. 10.1111/iju.14336 32776386

[B2] Bansal D. Chaturvedi S. R. M. Samit C. Anant K. (2022). Giant ureteral fibro-epithelial polyp: A rare but important differential of ureteral urothelial cell carcinoma. J. Clin. Urology 156, 515–518. 10.1177/2051415820931629

[B3] Chang C-W. Ou C-H. Yu C-C. Lo C-W. Tsai C-Y. Cheng P-Y. (2021). Comparative analysis of patients with upper urinary tract urothelial carcinoma in black-foot disease endemic and non-endemic area. BMC cancer 211, 80–89. 10.1186/s12885-021-07799-4 PMC7816491 33468084

[B4] Chen H. A. Liu S. Li X. J. Wang Z. Zhang C. Li F. Q. (2021). Clinical value of inflammatory biomarkers in predicting prognosis of patients with ureteral urothelial carcinoma. Beijing xue xue bao 2, 302–307. Yi xue ban= Journal of Peking University. Health Sciences 53. 10.19723/j.issn.1671-167X.2021.02.012 PMC8072417 33879902

[B5] Itami Y. Miyake M. Tatsumi Y. Gotoh D. Hori S. Morizawa Y. (2019). Preoperative predictive factors focused on inflammation-nutrition-and muscle-status in patients with upper urinary tract urothelial carcinoma undergoing nephroureterectomy. Int. J. Clin. Oncol. 245, 533–545. 10.1007/s10147-018-01381-y 30604161

[B6] Mori K. Resch I. Miura N. Laukhtina E. Schuettfort V. M. Benjamin P. (2021). Prognostic role of the systemic immune–inflammation index in upper tract urothelial carcinoma treated with radical nephroureterectomy: Results from a large multicenter international collaboration. Cancer Immunol. 9, 2641–2650. Immunotherapy 70. 10.1007/s00262-021-02884-w PMC8360829 33591412

[B7] Mounsif A. Cheriyan S. K. Foerster B. Shariat S. F. S. K. C. Charles C. P. Beat F. (2019). Optimal management of upper tract urothelial carcinoma: An unmet need. Curr. Treat. Options Oncol. 20 5, 40–11. 10.1007/s11864-019-0637-2 30937554

[B8] Nir K. F Matin P. S. Pierorazio P. M. Gore P. J. L. Ahmad S. Hu B. (2020). Primary chemoablation of low-grade upper tract urothelial carcinoma using UGN-101, a mitomycin-containing reverse thermal gel (OLYMPUS): An open-label, single-arm, phase 3 trial. lancet Oncol. 216, 776–785. 10.1016/S1470-2045(20)30147-9 32631491

[B9] Petros S. Pyrgidis N. Brookman-May S. Mykoniatis I. Karasavvidis T. Hatzichristou D. (2021). Does ureteral stenting increase the risk of metachronous upper tract urothelial carcinoma in patients with bladder tumors? A systematic review and meta-analysis. J. Urology 2054 (2021), 956–966. 10.1097/JU.0000000000001548 33284711

[B10] Ren S. Feng H. Bao Y. Yi W. Ou Y. Wang Y. (2021). Ureteral urothelial carcinoma with squamous cell carcinoma and sarcomatoid carcinoma differentiation: A case report. BMC Surg. 211, 96–6. 10.1186/s12893-021-01099-1 PMC7897460 33612111

[B11] Sebastiano N. Mazzone E. Preisser F. Tian Z. Mistretta F. A. Shariat S. F. (2019). Rates of lymph node invasion and their impact on cancer specific mortality in upper urinary tract urothelial carcinoma. Eur. J. Surg. Oncol. 45 (7), 1238–1245. 10.1016/j.ejso.2018.12.004 30563773

[B12] Takashi Y. Sato S. Kimura T. Iwatani K. Onuma H. Yanagisawa T. (2020). HER2 status in molecular subtypes of urothelial carcinoma of the renal pelvis and ureter. Clin. Genitourin. Cancer 184, e443–e449. 10.1016/j.clgc.2019.12.003 31983622

[B13] Teruo I. Matsuyama H. Komura K. Ibuki N. Fujimoto K. Shiina H. (2020). Tumor location based segmentation in upper-tract urothelial carcinoma impacts on the urothelial recurrence-free survival: A multi-institutional database study. Curr. Urol. 144, 183–190. 10.1159/000499240 PMC7810222 33488336

[B14] Wang J. Wang G. Shan H. Wang X. Wang C. Zhuang X. (2019). Gradiently degraded electrospun polyester scaffolds with cytostatic for urothelial carcinoma therapy. Biomater. Sci. 3, 963–974. Biomaterials. 10.1039/c8bm01317a 30569055

[B15] Wang Y. Liu H. Wang P. (2018). Primary sarcomatoid urothelial carcinoma of the ureter: A case report and review of the literature. World J. Surg. Oncol. 161, 77. 10.1186/s12957-018-1383-9 PMC5899376 29653574

[B16] Yang X. G. Jin M. S. Guo L. Zhu L. Qu L. M. (2019). Homolateral ureter primary small cell carcinoma and urinary tract epithelial carcinoma of the contralateral ureter and bladder after renal transplantation: Report of a case. Zhonghua bing li xue za zhi= Chin. J. pathology 489, 735–736. 10.3760/cma.j.issn.0529-5807.2019.09.018 31495101

[B17] Zhou W. Dang L. Chong C. Cheng X. Qin Y. Su W. (2022). Ureteral tumor with morphological features analogous to phyllodes tumor: A unique case with concomitant urothelial carcinoma. Diagn. Pathol. 171, 94–96. 10.1186/s13000-022-01277-6 PMC9784280 36564794

